# Applying Human-Centered Design to Develop Smartphone-Based Intervention Messages to Help Young Adults Quit Using E-Cigarettes and Cigarettes: A Remote User Testing Study

**DOI:** 10.2196/76503

**Published:** 2025-09-18

**Authors:** Thi Phuong Thao Tran, Christine Tran, Pamela M Ling, Lucy Popova, Nhung Nguyen

**Affiliations:** 1 School of Public Health, Georgia State University, Atlanta, GA, United States; 2 College of Health Sciences, VinUniversity, Hanoi, Vietnam; 3 HEARTY lab, University of California San Francisco, San Francisco, CA, United States; 4 Division of General Internal Medicine, Department of Medicine, University of California San Francisco, San Francisco, CA, United States; 5 UCSF Center for Tobacco Control Research and Education and Division of General Internal Medicine, 530 Parnassus Ave, San Francisco, CA, 94143, United States, 1 4154762265

**Keywords:** tobacco cessation, vaping, smoking, dual tobacco use, human-centered design, mHealth, smartphone intervention, young adults, mobile health

## Abstract

**Background:**

Despite the popularity of concurrent use of electronic cigarettes (e-cigarettes) and cigarettes (dual tobacco use) among young adults, few interventions address the cessation of both tobacco products. The application of a human-centered design (HCD) approach in the development of such interventions remains limited.

**Objective:**

This study used an HCD approach to develop smartphone-based intervention messages for dual tobacco cessation for young adults.

**Methods:**

Intervention messages were developed based on theories, cessation guidelines, existing messages, and our previous formative study. Three rounds of message testing were conducted asynchronously via an online platform with 35 young adults (18‐29 years old) who currently used both e-cigarettes and cigarettes and were motivated to quit either smoking or vaping in the next 6 months. In each round, a new sample of 10‐12 participants evaluated the messages individually. For the quantitative assessment, participants viewed and rated each message on a scale from 1 (“very low degree”) to 5 (“very high degree”) across 4 components: Comprehension (“This message is easy to understand”), Usefulness (“This message is useful for encouraging tobacco cessation”), Tone (“The language is clear and non-judgmental”), and Design (“The design is appealing”). For the qualitative assessment, participants used a platform-enabled feature to place markers on specific parts of messages they liked, disliked, or found confusing and then provided brief explanations for their feedback. Initial messages were assessed during the first 2 rounds of testing, and those with low mean scores were revised based on participants’ feedback and retested in the third round.

**Results:**

We found significant improvements in message ratings after refinement. The overall mean score increased from 3.6 (SD 0.4) to 4.6 (SD 0.2) (*P*<.001), using paired *t* tests. Specifically, the mean score of “Comprehension” improved from 4.0 (SD 0.5) to 4.9 (SD 0.2) (*P*<.001), the mean score of “Usefulness” increased from 3.0 (SD 0.6) to 4.4 (SD 0.4) (*P*<.001), the mean score of “Tone” increased from 3.8 (SD 0.6) to 4.8 (SD 0.2) (*P*<.001), and the mean score of “Design” increased from 3.4 (SD 0.48) to 4.4 (SD 0.3) *(P*<.001). The qualitative assessments highlighted design elements related to message liking, such as clear layout, minimalistic imagery, italicized quotes, and highlighted keywords. Conversely, design features related to message dislike included color shades, lengthy text, and confusing wording.

**Conclusions:**

This study demonstrated the use of HCD in developing smartphone-based intervention messages to support dual tobacco cessation among young adults. Integrating remote message testing improved the feasibility of rapid prototyping while enhancing the relevance and appeal of message content and design. Future interventions targeting emerging health behaviors among young adults may benefit from incorporating a remote testing method to efficiently gather user feedback and refine intervention messages in a timely manner.

## Introduction

Concurrent use of electronic cigarettes (e-cigarettes) and cigarettes in the past 30 days (dual tobacco use) is prevalent among US young adults aged 18 to 29 years. In 2021, among young adults who smoked cigarettes, 15%‐30% also used e-cigarettes; among young adults who used e-cigarettes, 30%‐67% also smoked cigarettes [1,2]. This dual-use behavior is associated with a higher risk of various health conditions, such as stroke, metabolic dysfunction, asthma, chronic obstructive pulmonary disease, and oral disease [3], and has been linked to an increased risk of nicotine dependence, subsequent substance use [4], and more difficulty with cessation [5]. Most young adults who use tobacco express a strong desire to quit and want to stop using all tobacco and nicotine products [6,7]. Yet, cessation interventions addressing both e-cigarettes and cigarettes remain limited [8].

Smartphone-based apps could offer cost-effective access to evidence-based cessation support for young adults [9], as over 95% of them own smartphones [10]. Of several available smartphone-based cessation apps, almost all were either for smoking cessation or vaping cessation [11], offered limited features for vaping cessation [12], and lacked rigorous development and testing [13-15]. Furthermore, existing apps typically adopt a “one-size-fits-all” approach and often fail to incorporate meaningful input from end users early in the development process [16]. This limited evidence on effective strategies addressing dual tobacco use among young adults highlights the need for developing new intervention messages addressing the use of both e-cigarettes and cigarettes among young adults [8,17].

A human-centered design (HCD) approach can be used to develop a smartphone app intervention that meets a variety of individual cessation needs [18]. HCD is defined as *“*an approach to systems design and development that aims to make interactive systems more usable by focusing on the use of the system and applying human factors/ergonomics and usability knowledge and techniques*”* [19]. Its systematic approach engages with and prioritizes the needs and preferences of end users in the development of an intervention, ensuring that interventions are likely to be used effectively in a real-world context [18]. We used the IDEAS framework (including 4 stages: Integrate, DEsign, Assess, and Share, described in the next paragraph), which integrates behavioral theory, design thinking, and intervention evaluation and dissemination into systematic guidance for the development of effective interventions [20]. This framework has guided the development of previous substance use interventions, including tobacco smoking cessation [21-23]. There are 10 phases (Empathize, Specify, Ground, Ideate, Prototype, Gather, Build, Pilot, Evaluate, and Share) across the 4 stages. Although HCD is increasingly used in mobile health interventions, there is limited published research on how the HCD approach is applied to developing interventions for young adult dual tobacco cessation [24].

By applying the HCD approach, the first 3 phases in the “*Integrate”* stage (empathizing with the target users*,* specifying targets for future interventions*,* and grounding findings in behavioral theories) were conducted in our previous formative research using in-depth interviews with young adults to elicit needs and preferences for dual tobacco cessation interventions [6]. This formative work led to our “*Design”* stage, in which we aim to ideate intervention strategies, prototype intervention messages, gather user feedback, and build an intervention prototype that includes smartphone-based intervention messages for dual tobacco cessation among young adults. The developed intervention prototype will then be used in the next stage, *“Access,”* where we plan to pilot and evaluate its efficacy in a randomized controlled trial (RCT). Focusing on the “*Design*” stage, this study aims to describe our process for ideating, prototyping, and refining intervention message mockups through iterative feedback from young adults. Our remote HCD approach may serve as a methodological model for message development and remote testing in future digital health interventions.

## Methods

### Design

This study is the second phase of a project aiming to develop a smartphone-based intervention targeting dual tobacco cessation among young adults. In the first phase, we conducted a needs assessment study to understand young adults’ desires and preferences for cessation of both e-cigarette vaping and cigarette smoking [6]. In the second phase (ie, this study), we developed intervention messages for use in the smartphone-based intervention, which will be evaluated in an RCT in the next phase.

### Participants

Participants were recruited through targeted advertisements on Instagram linking to the study website. Eligible participants were young adult men and women (aged 18 to 29 years) who: (1) speak English, (2) own a smartphone, (3) report use of both cigarettes and e-cigarettes at least weekly during the past month [6], and (4) intend to quit either smoking or vaping in the next 6 months. Overall, 35 participants were enrolled in message testing.

### Message Development

**Figure 1.**
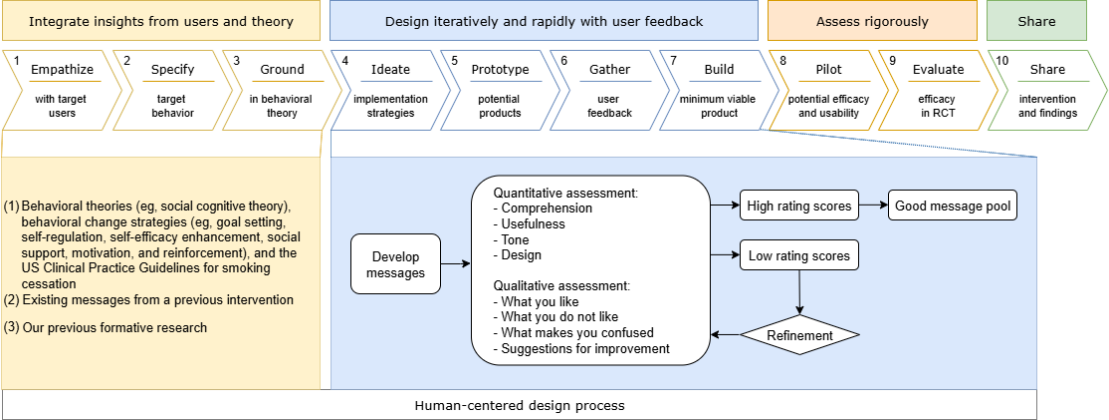
Human-centered design approach in the development of tobacco cessation messages for young adults who use both electronic cigarettes and cigarettes. RCT: randomized controlled trial.

The HCD approach in the development of tobacco cessation messages is displayed in Figure 1. The message development was guided by behavioral theories (eg, Social Cognitive Theory), behavioral change strategies (eg, goal setting, self-regulation, self-efficacy enhancement, social support, motivation, and reinforcement) [25], and the US Clinical Practice Guidelines for smoking cessation [26]. The message content was also based on existing messages in a previous intervention that effectively increased smoking abstinence among young adults [26]. We further refined message content to specifically target young adult dual tobacco users’ needs and preferences determined previously in our formative research, including providing cessation support to quit one prioritized product while preventing switching between 2 products, and emphasizing unique facilitators and barriers to quitting each product (eg, unpleasant smell of cigarettes facilitating smoking cessation and accessibility of e-cigarettes hindering vaping cessation) [6].

To fit with smartphone interfaces, we then designed 90 mockups of the messages. We added design elements (eg, color and style of text, graphics, and pictures) to the text messages to enhance visual aesthetics and user engagement.

### Message Testing and Refinement

The testing was conducted remotely via Recollective (Recollective Inc), a platform for conducting user experience research. Each participant logged into the platform via phone or computer to complete the message rating tasks at a time convenient for them. This flexible and self-paced approach with asynchronous features could enhance accessibility to the testing process. We conducted 3 rounds of testing, with the first 2 rounds assessing the developed messages (prerefinement) and the third round retesting the refined messages (postrefinement). To enhance the representativeness of the target users and gather diverse insights, we included a new sample of 10‐12 young adults in each round. This approach also minimized participant fatigue and disengagement through repeated rounds of testing. We provided participants with a video with detailed instructions to facilitate their message assessment. Participants evaluated the messages individually, with no access to others’ assessments. Each message rating task took 3‐5 minutes to complete. To avoid overburdening participants and allow sufficient time to complete all message ratings, we asked participants to evaluate 45 messages within 2 weeks in each round of testing (a total of 2 testing rounds). Our team monitored participants’ progress daily and sent reminders to ensure the message evaluation was completed within the predefined timeframe.

**Figure 2.**
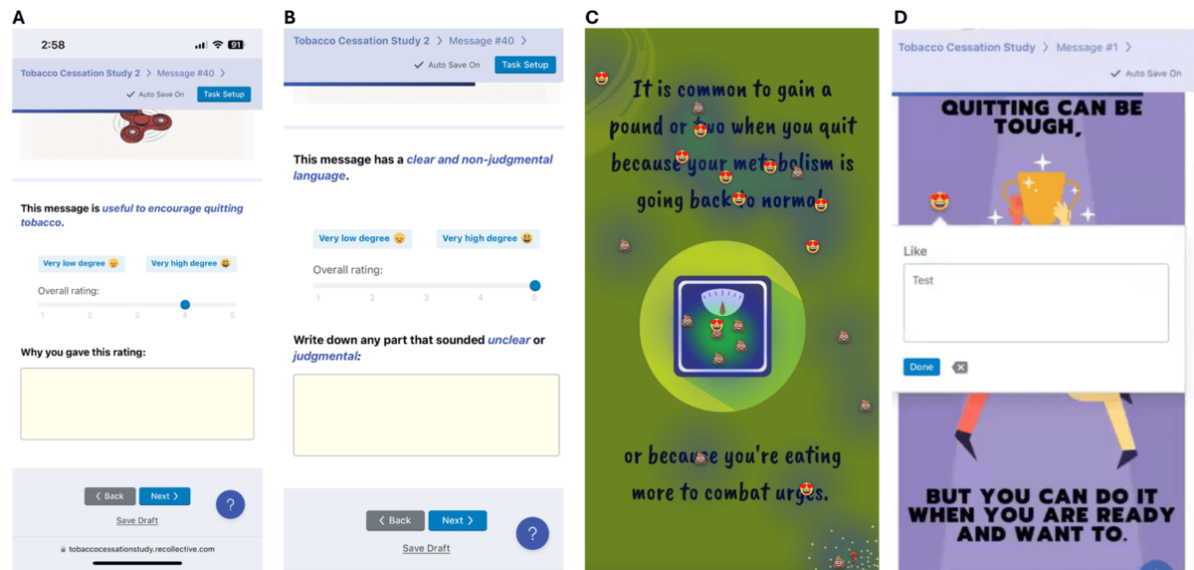
Screenshots illustrating the testing process: (A and B) examples of component rating, (C) markers indicating whether participants liked, disliked, or found messages confusing, and (D) a text box for providing brief explanations of their specific reasons. The icons on the original message indicate the specific parts that participants liked (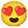), disliked (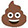), or found confusing (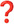).

Messages were evaluated using both quantitative and qualitative assessments. For the quantitative assessment, participants viewed and rated each message on a scale from 1=“very low degree” to 5=“very high degree” across 4 components, with 1 question for each component: Comprehension (ie, “This message is easy to understand”), Usefulness (ie, “This message is useful for encouraging tobacco cessation”), Tone (ie, “The language is clear and non-judgmental”), and Design (ie, “The design is appealing”) [27,28], together with optional text box provided for participants to explain their ratings (Figure 2A and 2B). For the qualitative assessment, participants could place an unlimited number of markers on specific parts of the messages they liked, disliked, or found confusing, without seeing others’ markers (Figure 2C). For each marker placed, participants were asked to type in a pop-up text box to briefly explain their specific reasons for liking, disliking, or confusion (Figure 2D).

All 90 developed messages were revised based on participants’ feedback. Due to time and resource constraints for rapid testing, we retested only 35 out of 90 messages with low rating mean scores (eg, a mean score of less than 4 out of the maximum score of 5) in the subsequent round, while the remaining messages with high rating scores were also revised but not retested [24].

We encountered a small number of open-ended comments that were ambiguous or lacked sufficient context for accurate interpretation. Our team carefully reviewed these comments and collaboratively discussed potential meanings. When a comment could not be reasonably interpreted through consensus discussion, it was excluded from the message revision to avoid potential misrepresentation. These exclusions were minimal and ensured that only responses with sufficient clarity informed the message refinement.

### Data Analysis

For the qualitative analysis of participants’ comments, we summarized their feedback on design features, confusing content, notes on usefulness and tone, and suggestions for message improvement.

To evaluate the overall improvement of the 35 retested messages, we compared the mean scores across the 4 components (Comprehension, Usefulness, Tone, and Design) and the overall score before and after refinement. Messages that initially had high scores and did not require refinement were excluded from the analysis. Paired *t* tests were conducted to examine whether the postrefinement mean scores were significantly higher than the prerefinement mean scores. The quantitative data analysis was performed using STATA software version 18 (StataCorp LLC).

### Ethical Considerations

The study was approved by the Institutional Review Board at the University of California, San Francisco (no. 352216). All participants were provided with the study information and completed electronic informed consent on the study website. An identification number was assigned to each participant to maintain anonymity, and all data were deidentified. Participants received a US $100 e-gift card for their participation.

## Results

### Participants’ Characteristics

The demographic characteristics of study participants are shown in Table 1. The study sample had a mean age of 22 years (SD 3). In total, 20 out of 35 participants (57%) were female and 26 participants (74%) were not Hispanic, Latino, or of Spanish origin. Regarding race, the largest group was White (n=20, 57%), followed by multiracial individuals (n=6, 17%), Asian (n=4, 11%), and Black or African American (n=3, 9%). In addition, 27 out of 35 participants (77%) were currently enrolled in college.

**Table 1.** Demographic characteristics of study participants (N=35).

Characteristics	Value
Age (years), n (%)	
Min-max	18‐29
Mean (SD)	22 (3)
Gender, n (%)	
Female	20 (57)
Male	15 (43)
Ethnicity, n (%)	
Hispanic, Latino, or Spanish	9 (26)
Not Hispanic, Latino, or Spanish	26 (74)
Race, n (%)	
White	20 (57)
Asian (Chinese, Vietnamese, Asian Indian)	4 (11)
Black or African American	3 (9)
American Indian or Alaska Native	1 (3)
Middle Eastern	1 (3)
Multiracial	6 (17)
Education attainment, n (%)	
Currently in college	27 (77)
Currently in graduate school	2 (6)
Currently in professional or technical school	1 (3)
Not attending school	5 (14)

### Results of Message Development

To address dual tobacco cessation, we developed messages that encourage quitting all tobacco and nicotine products by using terms “any tobacco” or “nicotine.” Another strategy was to develop messages that address shared barriers (eg, craving) and facilitators (eg, social support and rewarding) for quitting both e-cigarettes and cigarettes, as identified in our previous formative research. In addition, our previous study indicated that users usually did not attempt to quit both tobacco products simultaneously but instead prioritized quitting one before the other. Therefore, in addition to promoting dual tobacco cessation, we also developed single-product cessation messages. This tailored approach allows message delivery to align with an individual’s preferred quitting sequence, which can be implemented in the next phase. For instance, if a user prioritized quitting smoking, they would receive more messages focused on smoking cessation while still being exposed to messages encouraging the cessation of all tobacco and nicotine products in our future RCT. Moreover, we also developed messages to prevent switching between e-cigarettes and cigarettes. Multimedia Appendix 1 provides examples of these messages before refinement, including those promoting dual tobacco cessation, addressing shared barriers and facilitators to quitting both products (eg, craving management, social support, and rewarding), targeting single-product cessation, and discouraging switching between e-cigarettes and cigarettes.

### Results of Message Testing and Refinement

#### Overview

After each round of testing, we revised the messages based on participants’ feedback. One key challenge in this refinement process was balancing the diverse needs and preferences of young adults. Participants often provided multiple, sometimes conflicting, suggestions for improving the messages, making it difficult to determine the best approach to fully address the diverse feedback. To address this issue, our team balanced their needs with practical feasibility and resource restraints. In some cases, we created multiple versions of messages tailored to different subgroups. Multimedia Appendix 2 shows an example of two revised versions of a message promoting exercise to manage nicotine cravings, tailored separately for males and females.

#### Quantitative Results

To meet the rapid timeline of iterative testing cycles, we prioritized refining the 35 messages with low ratings and retested them in the subsequent round. Multimedia Appendix 3 presents average scores for the 35 retested messages before and after refinement, covering 4 components. All 4 components showed significant improvement after refinement: Comprehension increased from 4.0 (SD 0.5) to 4.9 (SD 0.2), Usefulness rose from 3 (SD 0.6) to 4.4 (SD 0.4), Tone increased from 3.8 (SD 0.6) to 4.8 (SD 0.2), and Design increased from 3.4 (SD 0.48) to 4.4 (SD 0.3). Overall, the mean score increased significantly after refinement, rising from 3.6 (SD 0.4) to 4.6 (SD 0.2). All paired *t* tests showed statistically significant results with *P*<.001.

#### Qualitative Results

Overall, based on the participants’ comments during the message testing, the design features that participants liked the most included a clear layout, minimalistic imagery, italicized quotes*,* and highlighted keywords. The design features of message disliking included color shades, lengthy text, and confusing words. Given the large number of refined messages, below we provide 2 examples illustrating how specific messages were revised based on participants’ feedback.

**Figure 3.**
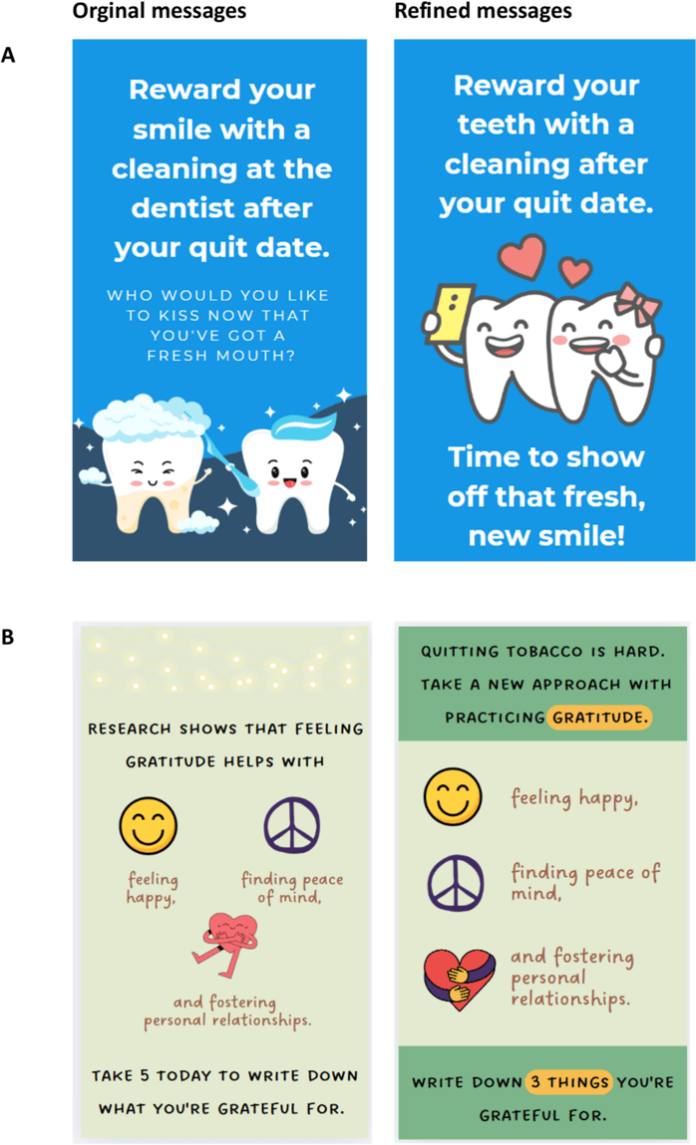
Two examples of messages before and after refinement based on participants’ feedback.

Figure 3A provides an example of a message encouraging self-reward after quitting. While many participants liked illustrations and colors, some found the original messages confusing, with one stating, *“a bit confused as to why the bottom half of the left tooth looks like cheese.”* To address this, we revised the graphic by removing the yellow streaks and replacing them with smiley teeth illustrations to maintain consistency across messages. In addition, we removed the word *“dentist,”* as many participants expressed their dislike of this word. Some participants also found the message somewhat unclear and *“seems kind of out of left field, not extremely relevant,”* and suggested *“change the text to make it more convincing.”* In response, we revised the bottom text to be more direct and affirmative, aligning with a common theme of the message.

Figure 3B provides an example of a message promoting gratitude and emotional well-being as supportive strategies for tobacco cessation. Some participants expressed that the message seemed unrelated, stating that *“not see how this relates to vaping,” “not specific to tobacco,” “not sure if they are trying to convince people to give up tobacco,”* and *“not sure what this message is trying to address.”* To strengthen its relevance to tobacco cessation, we added an element acknowledging that “*quitting tobacco is hard*.” In addition, given that messages telling people to be grateful can come off wrong to some people, we refined it as a coping strategy for quitting. The bottom part was refined to be more specific and actionable, encouraging participants to identify 3 things they are grateful for.

## Discussion

### Principal Results

This study showcases an HCD approach to develop messages for a smartphone-based intervention to support young adults’ dual tobacco cessation. As an early stage of intervention development, we built a low-cost and low-fidelity prototype of the intervention (mockups of intervention messages) that could be developed rapidly to gather feedback from target users early and often, allowing exploration of alternative solutions before investing significant resources in any one particular or potentially suboptimal solution. We found that the HCD approach significantly improved the comprehension, usefulness, tone, and design of the intervention messages, which can ultimately increase the intervention’s real-world impact and end-user engagement. As a methodological example of remote message testing, our study demonstrated the feasibility of engaging young adults in remote user experience testing without the need for face-to-face interactions, which can speed up the intervention development process while saving research costs. Another notable contribution of this study is to develop intervention messages specifically for dual tobacco cessation among young adults—a critical gap in the literature.

In this study, we applied the HCD principles to translate evidence-based tobacco cessation treatment into a real-world solution. Aligned with a traditional approach, we initially developed core message content based on a comprehensive evidence base, combining best practices for tobacco treatment, behavioral theories, behavior change techniques, previous interventions, and our formative needs assessment. Traditional message development methods are typically content-focused and expert-driven, which could overlook the lived experiences, preferences, and contextual needs of the end users. Rooted in empathy-driven focus, our HCD approach addresses these issues by prioritizing the preferences of end users and integrating design strategies that enhance the engagement and usability of the core content. Particularly, our participants’ iterative feedback revealed that messages could be made more appealing by incorporating a clear layout, minimalistic imagery, italicized quotes, and highlighted keywords while avoiding lengthy text and confusing wording. This is especially important for smartphone apps, as well-designed message interfaces can significantly influence how young adults perceive messages’ clarity, appeal, and the overall effectiveness in supporting tobacco cessation.

### Comparison with Prior Work

Unlike previous smartphone app interventions that primarily focused on single-product smoking cessation (eg, cigarette use) and were often grounded in single behavioral change theory, our study used a more robust approach to intervention design. We integrated multiple behavioral theories or techniques and clinical guidance with users’ needs and preferences. This user-centered design process ensured that the messages were not only scientifically evidence-based but also relevant and engaging in a real-world context. Previous studies used an HCD approach to design mHealth apps for smoking cessation among pregnant women [29], people with serious mental illness [30], and people with HIV [29-33]. Consistent with our findings, user-centered design and usability testing in these studies helped align the interventions with users’ needs for smoking cessation. Moreover, this approach also enhances the ability to create multiple tailored message versions (eg, for males and females), enhancing the personalization of interventions, which is an important direction highlighted in previous mHealth research [34].

A key contribution of this study is the use of remote quantitative and qualitative assessments from target users, offering greater flexibility and accessibility than traditional in-person feedback collection. This approach enabled participants to provide detailed input not only on content but also on specific design elements, such as colors, symbols, and other visual features. The entire process of message testing was conducted remotely, with the self-guided video instruction provided beforehand, which allowed participants to complete the tasks independently. Unlike prior studies that relied heavily on researcher-facilitated interviews or focus groups, our method reduced the need for real-time involvement from the research team, minimizing the time and resource burden on both researchers and participants. This low-cost, asynchronous approach also enhances access to user testing and may facilitate more open and honest feedback as participants provide responses privately, potentially reducing social desirability bias often present in interviews and the “groupthink” effect commonly observed in focus groups [35]. Overall, our remote testing method presents a promising and scalable methodology for future message development efforts targeting emerging health behaviors among young adults.

### Future Research

The final message set will be implemented in the next phase, “*Assess,”* in which we will deliver the intervention via smartphone app and evaluate its efficacy and effectiveness on dual tobacco cessation among young adults. Past research highlighted the need to investigate important mediators of smartphone interventions’ success, such as psychological empowerment (the intervention helping one find ways to think and behave differently), hedonic well-being (the intervention making one happier), and inspiration (the intervention motivating one to be a person they want to be) [36]. These perceptions are influenced by the content of the intervention and are, in turn, predictive of behavior, such that psychological empowerment and hedonic well-being are positively, but inspiration is negatively, associated with quitting and reducing smoking [36]. Thus, in future studies, it would be worthwhile to not only assess the impact of the intervention on tobacco cessation, but also examine these and other potential mechanisms of impact.

### Limitations

First, the participants in our study may not fully represent the broader young adult population, and recruiting through social media may have restricted our reach to more diverse groups. This may reflect the perspectives and experiences of White younger individuals who are more digitally connected, limiting the breadth of feedback for the intervention messages and the generalizability of our findings to the broader target population. Therefore, future studies could consider alternative recruitment strategies to ensure a more representative sample. Second, some open-ended comments from participants were unclear, which posed challenges for interpretation. Finally, since our repeated testing involved different participant samples, significant differences in quantitative assessments may reflect not only improvements to the messages but also variations in sample characteristics. While engaging different groups allowed us to capture feedback from more young adults, this approach may introduce the possibility of confounding due to between-sample differences. To mitigate this limitation, future studies may consider a longitudinal design in which the same participants assess messages over time, allowing for greater control over individual-level variability and clearer attribution of changes to message revisions.

### Conclusions

In conclusion, this study offers valuable insights into using a remote HCD approach to develop smartphone-based intervention messages for young adults aiming to quit e-cigarettes and cigarettes. The use of self-instructional videos and asynchronous feedback collection enhanced the accessibility and feasibility of remote message testing. This approach improved message comprehension, usefulness, tone, and design. These findings can inform future message development and remote user testing efforts for tobacco cessation. Moreover, our HCD process is adaptable to other behavioral topics, populations, delivery formats (eg, video and audio), and platforms (eg, social media). Future research can build on this work to optimize engagement and effectiveness in digital health interventions targeting health behavior changes among young adults.

## Supplementary material

10.2196/76503 Multimedia Appendix 1 Examples of messages before refinement in this study.

10.2196/76503 Multimedia Appendix 2 Examples of messages tailored to males and females.

10.2196/76503 Multimedia Appendix 3 Average scores for the 35 retested messages before and after refinement, covering 4 components: Comprehension, Usefulness, Tone, and Design.

## Abbreviations

e-cigarette: electronic cigarette
HCD: human-centered design
RCT: randomized controlled trial
