# Supplementary material for: Applying Human-Centered Design to Develop Smartphone-Based Intervention Messages to Help Young Adults Quit Using E-Cigarettes and Cigarettes: A Remote User Testing Study
Source: JMIR Hum Factors. 2025 Sep 18;12:e76503. doi: 10.2196/76503 (PMC12445061; doi:10.2196/76503)
Supplement: Multimedia Appendix 1 [file humanfactors-v12-e76503-s001.docx]

Multimedia Appendix #1 - Examples of messages before refinement in this study.

| **Messages promoting dual tobacco cessation** |
| --- |
| 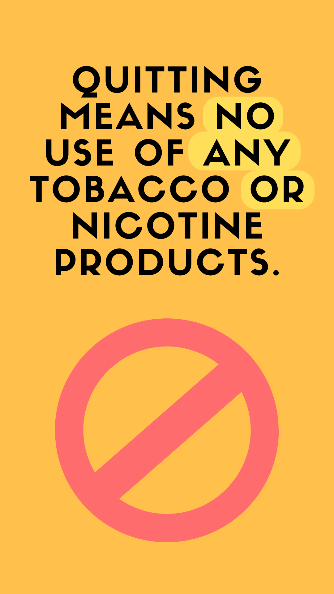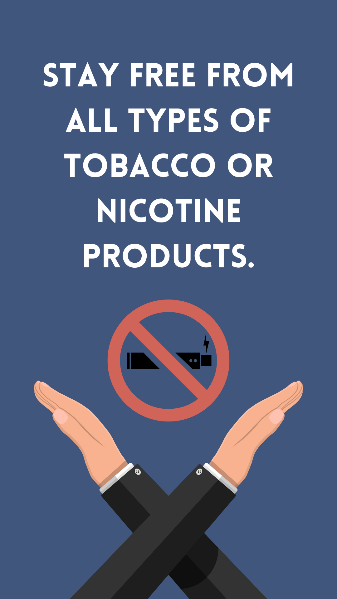 |
| **Messages addressing shared barriers and facilitators for quitting both products** |
| Craving management  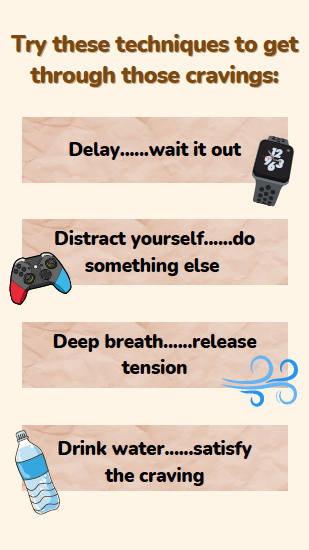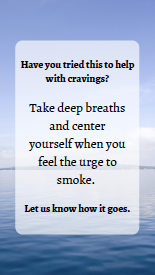 |
| Social support  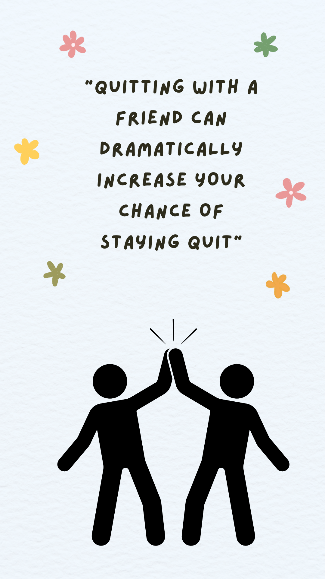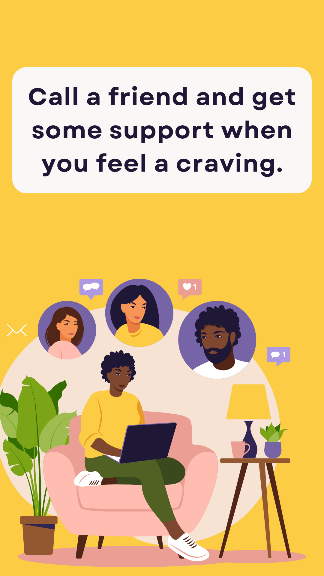 |
| Rewarding  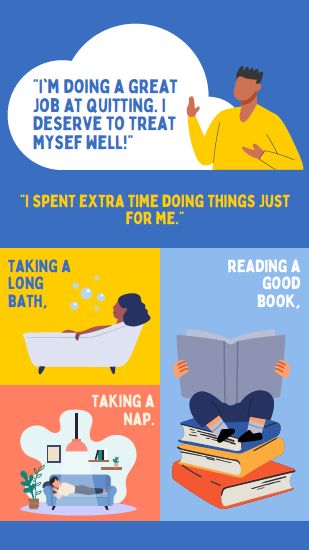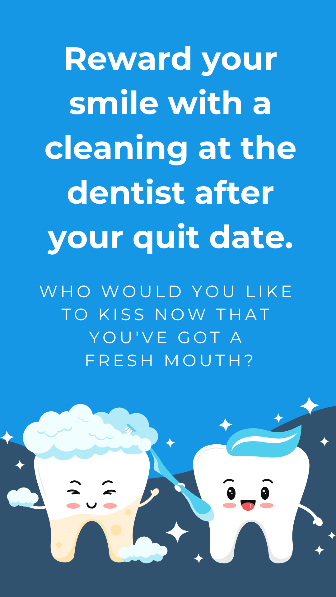 |
| **Messages targeting single-product cessation** |
| 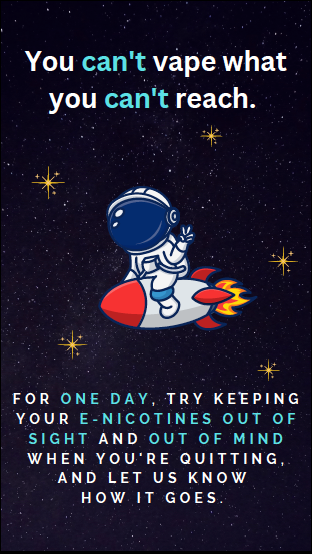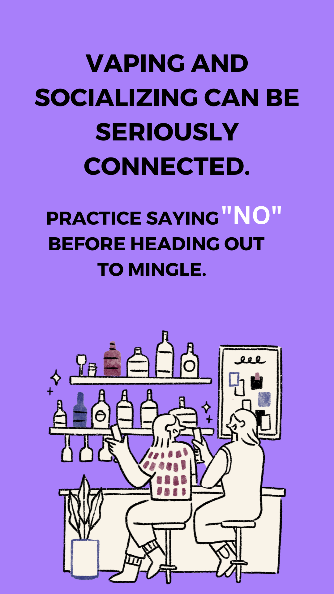 |
| **Messages discouraging switching between e-cigarettes and cigarettes.** |
| **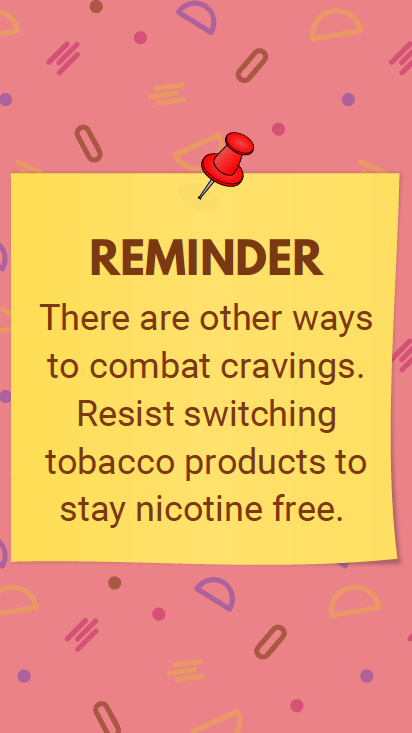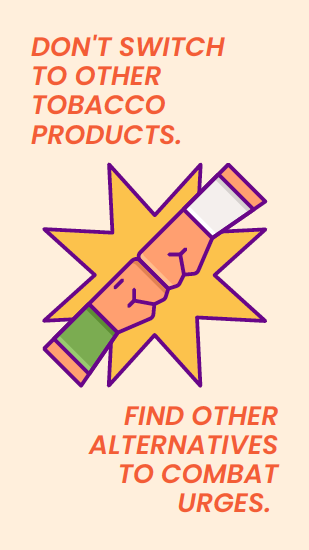** |
