# Supplementary figures and images for: Applying Human-Centered Design to Develop Smartphone-Based Intervention Messages to Help Young Adults Quit Using E-Cigarettes and Cigarettes: A Remote User Testing Study
Source: JMIR Hum Factors. 2025 Sep 18;12:e76503. doi: 10.2196/76503 (PMC12445061; doi:10.2196/76503)

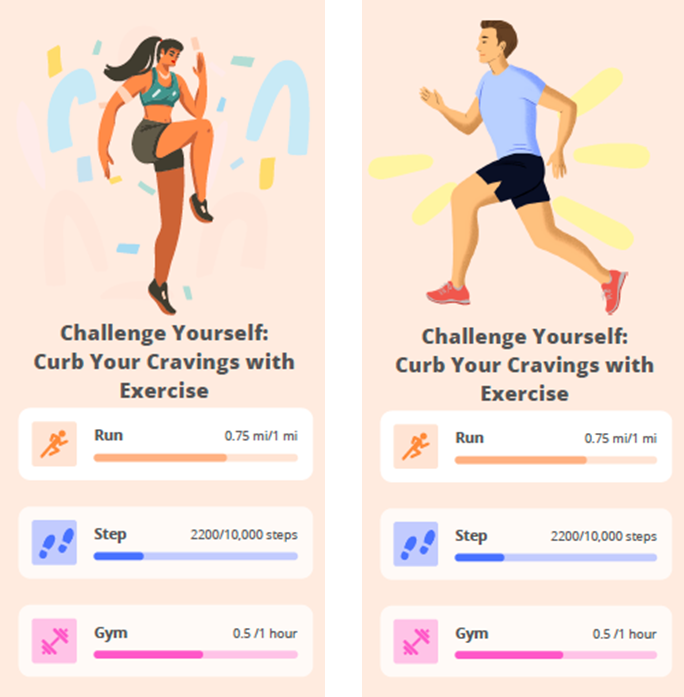

Supplement: Multimedia Appendix 2 [file humanfactors-v12-e76503-s002.png]

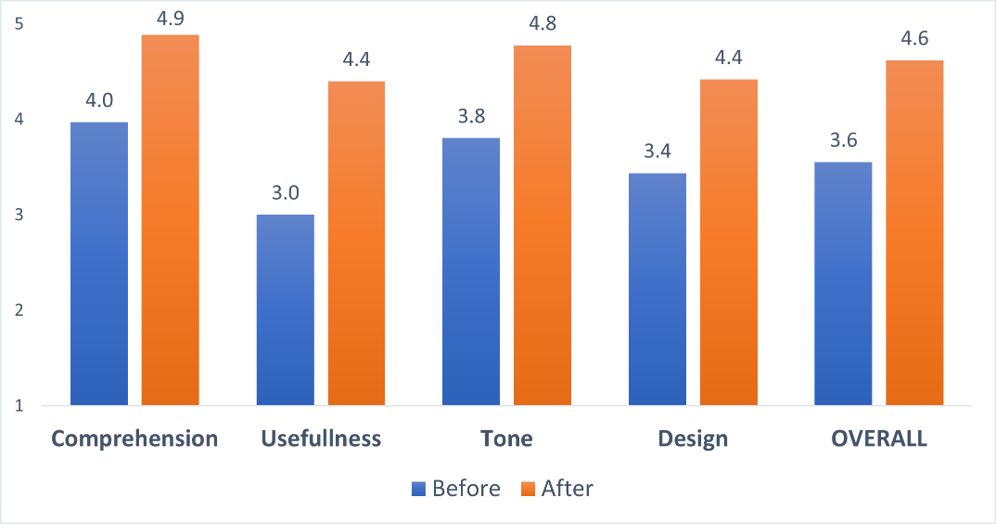

Supplement: Multimedia Appendix 3 [file humanfactors-v12-e76503-s003.png]
